# Supplementary material for: A deeper look at carrier proteome effects for single-cell proteomics
Source: Commun Biol. 2022 Feb 22;5:150. doi: 10.1038/s42003-022-03095-4 (PMC8863851; doi:10.1038/s42003-022-03095-4)
Supplement: Supplementary file 2 — Supplementary information [file 42003_2022_3095_MOESM2_ESM.pdf]

# A deeper look at carrier proteome effects for single-cell proteomics

Zilu Ye[1], Tanveer S Batth[1], Patrick Rüther[1], Jesper V. Olsen[1*]

[1] Novo Nordisk Foundation Center for Protein Research, Faculty of Health and Medical Sciences, University of Copenhagen, Copenhagen, Denmark

[*]To whom correspondence should be addressed. E-mail: jesper.olsen@cpr.ku.dk

**Supplementary Figures**

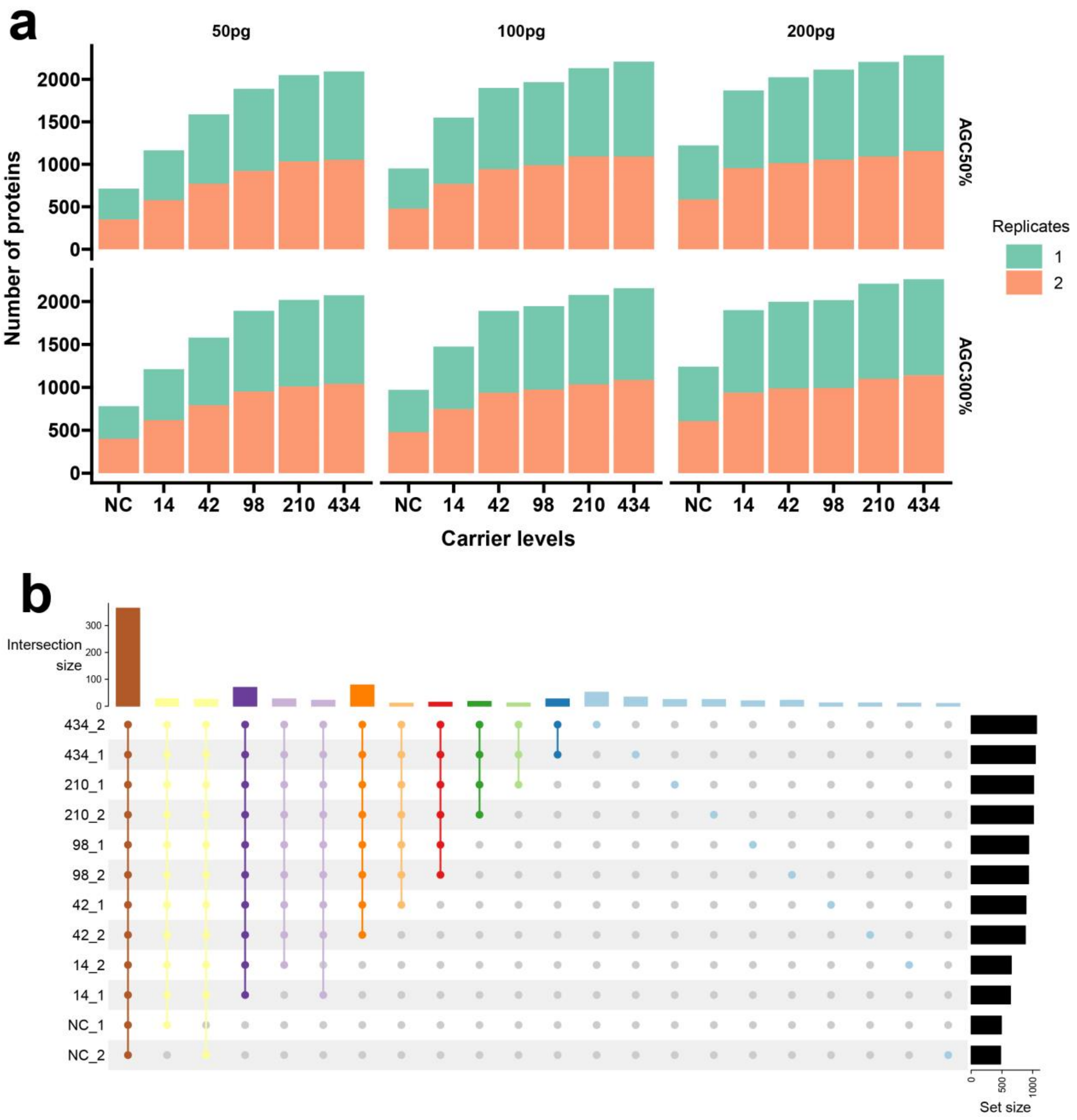


**Supplementary Figure 1 | a)** Number of human proteins identified in samples with H-only as carrier proteomes and without carrier proteome. **b)** UpSet plot of the identified proteins in the same samples. NC: no carrier.

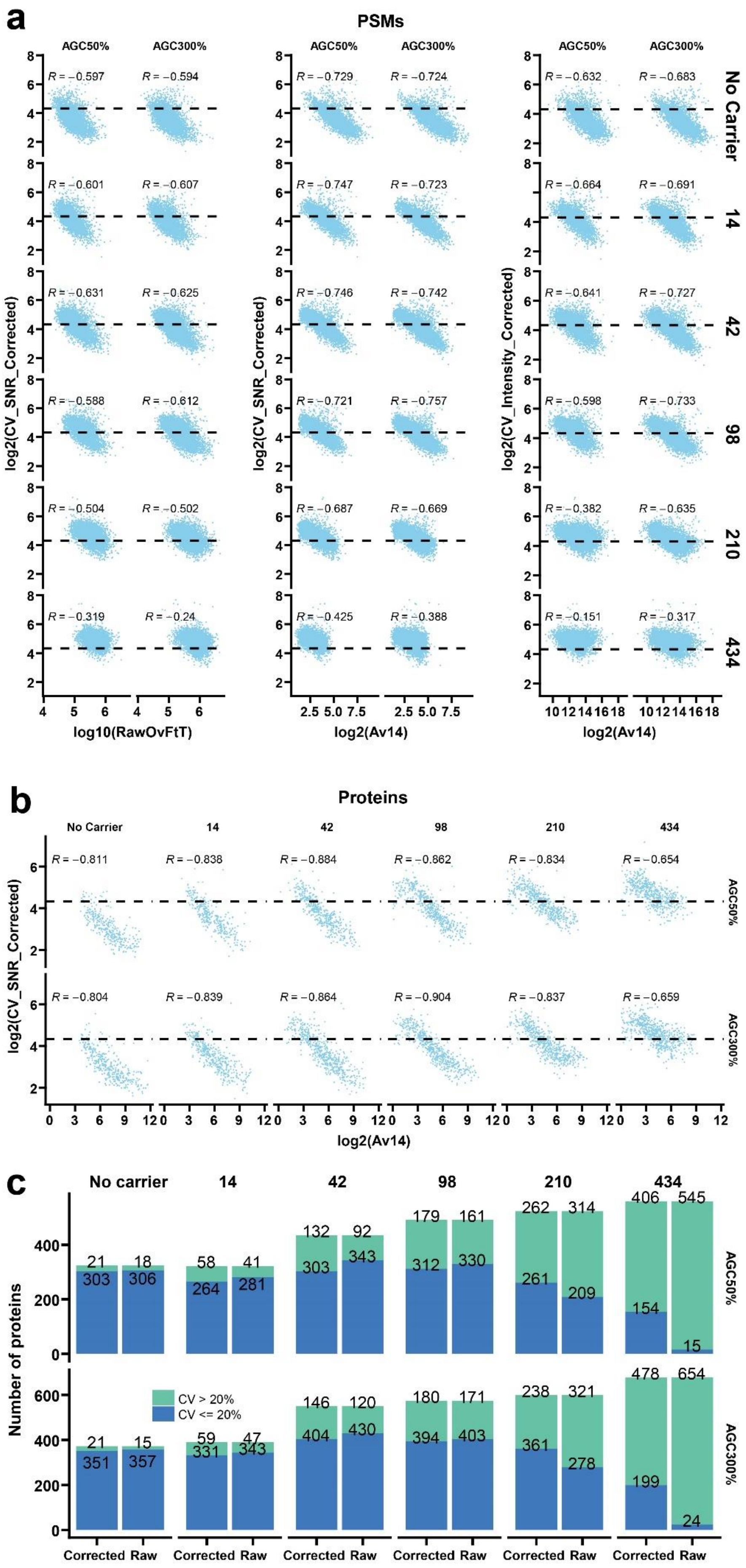

a
PSMs
AGC50%
AGC300%
No Carrier
14
42
98
210
434
log2(CV_SNR_Corrected)
log2(CV_Intensity_Corrected)
log10(RawOvFtT)
log2(Av14)
b
Proteins
No Carrier
14
42
98
210
434
AGC50%
AGC300%
log2(CV_SNR_Corrected)
log2(Av14)
c
No carrier
14
42
98
210
434
Number of proteins
CV > 20%
CV <= 20%
AGC50%
AGC300%
Corrected
Raw

**Supplementary Figure 2 | Relationship between average abundances of 14 single cell channels (Av14) and CV values.** **a**) Distribution of CV values in identified PSMs. **Left panel**) relationship between RawOvFtT and CV values calculated from impurity corrected SNR; **middle panel**) relationship between Av14 and CV values calculated from impurity corrected SNR; **right panel**) relationship between Av14 and CV values calculated from impurity corrected intensities. **b**) Distribution of Av14 calculated from impurity corrected SNR and CV values in identified proteins from human proteins. **c**) Number of identified proteins with CV > 20% and CV <= 20% using either raw or corrected SNR. Samples with the following settings were selected: no carrier and carrier proteome as HEY; AGC50% and AGC300%; 50pg per single cell channel; replicate 2. Av14 values lower than 1 were excluded in the analysis. Spearman's rank correlation coefficient was used as a measure of rank correlation in both **a**), **b**) and **c**). The dash lines indicate CV value of 20%.

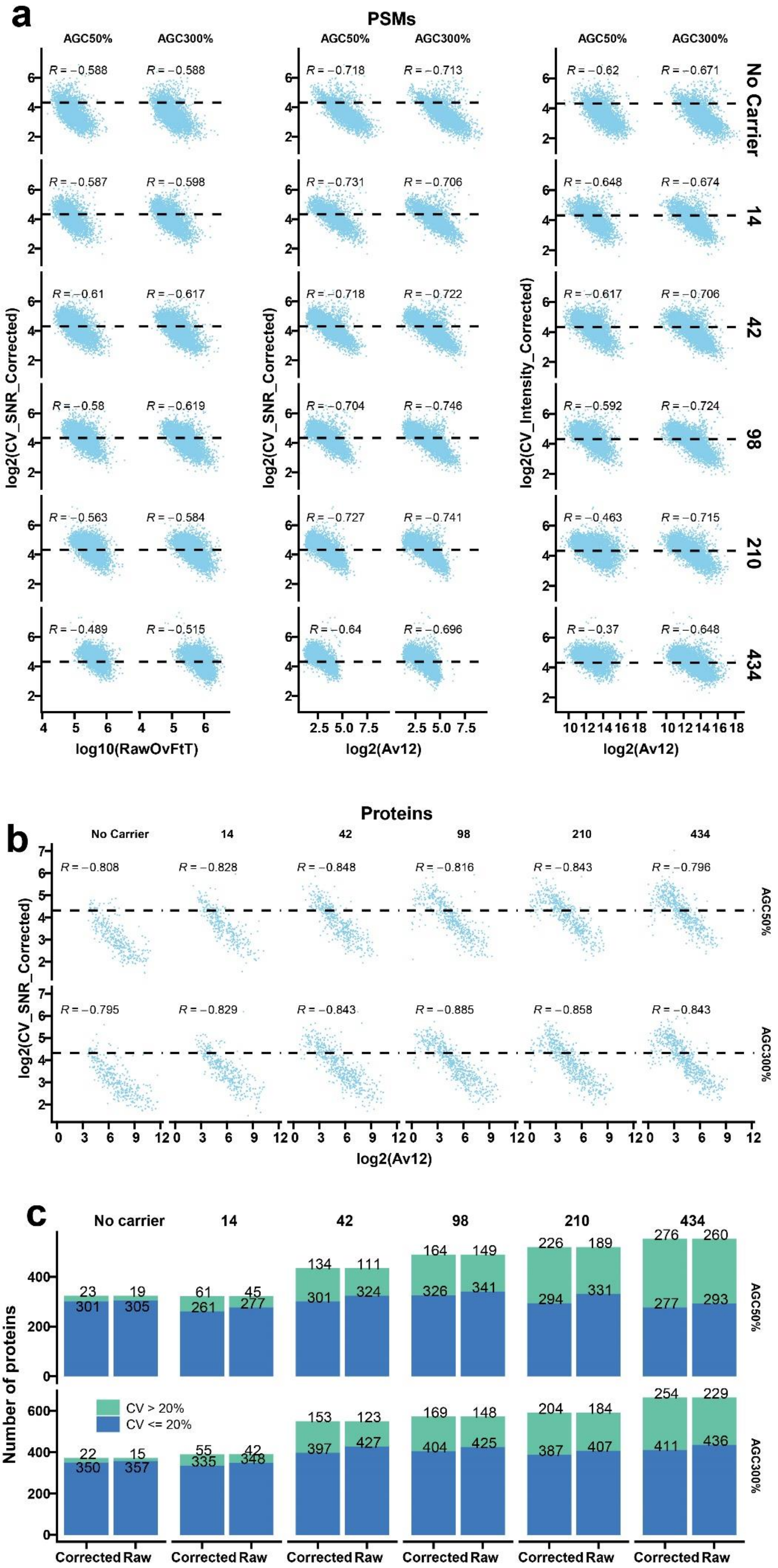
a
PSMs
AGC50%
AGC300%
No Carrier
14
42
98
210
434
log2(CV_SNR_Corrected)
log2(CV_Intensity_Corrected)
log10(RawOvFtT)
log2(Av12)
b
Proteins
No Carrier
14
42
98
210
434
log2(CV_SNR_Corrected)
log2(Av12)
AGC50%
AGC300%
c
No carrier
14
42
98
210
434
Number of proteins
CV > 20%
CV <= 20%
Corrected
Raw
AGC50%
AGC300%

**Supplementary Figure 3 | Relationship between average abundances of 12 single cell channels (Av12, 127N and 128C excluded) and CV values.** **a**) Distribution of CV values in identified PSMs. **Left panel**) relationship between RawOvFtT and CV values calculated from impurity corrected SNR; **middle panel**) relationship between Av12 and CV values calculated from impurity corrected SNR; **right panel**) relationship between Av12 and CV values calculated from impurity corrected intensities. **b**) Distribution of Av12 calculated from impurity corrected SNR and CV values in identified proteins from human proteins. **c**) Number of identified proteins with CV > 20% and CV <= 20% using either raw or corrected SNR. Samples with the following settings were selected: no carrier and carrier proteome as HEY; AGC50% and AGC300%; 50pg per single cell channel; replicate 2. Av12 values lower than 1 were excluded in the analysis. Spearman's rank correlation coefficient was used as a measure of rank correlation in both **a**), **b**) and **c**). The dash lines indicate CV value of 20%.

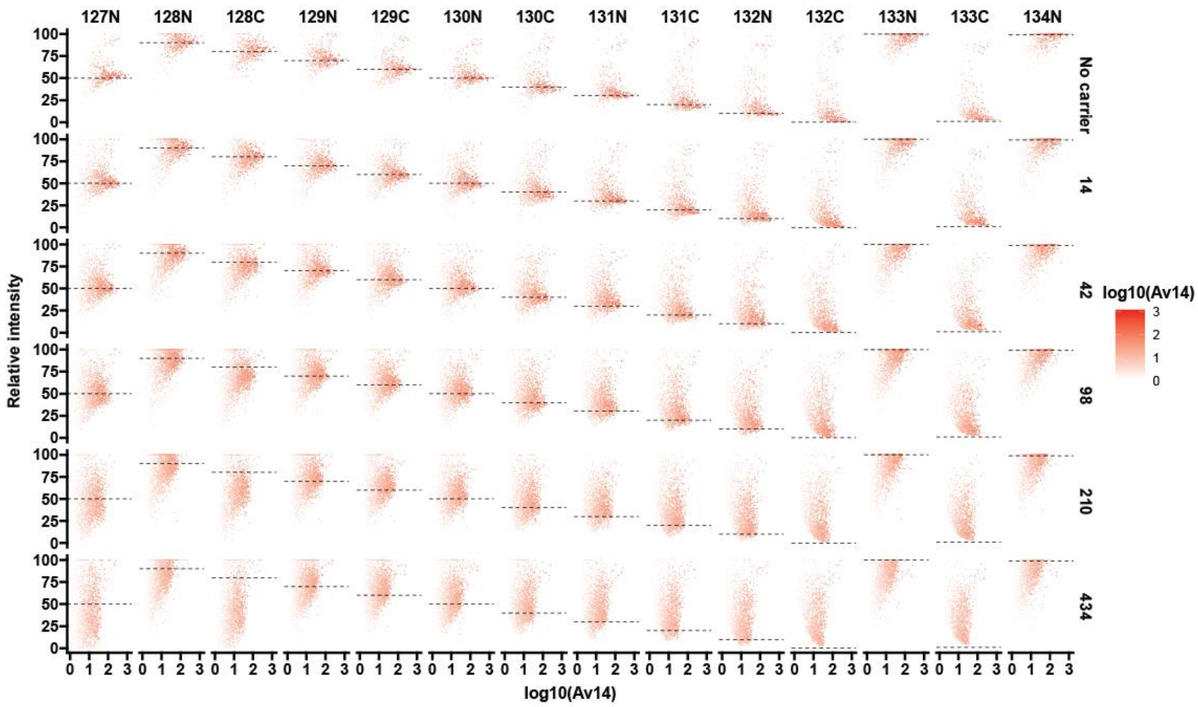


**Supplementary Figure 4 | Relative intensity of reporter ions in yeast peptides.** PSMs with *Parent intensity fraction* >= 0.98 and Av14 >= 1 were kept. Samples with the following settings were selected: no carrier and carrier proteome as EY. Impurity corrected SNR were used for the reporter ions.

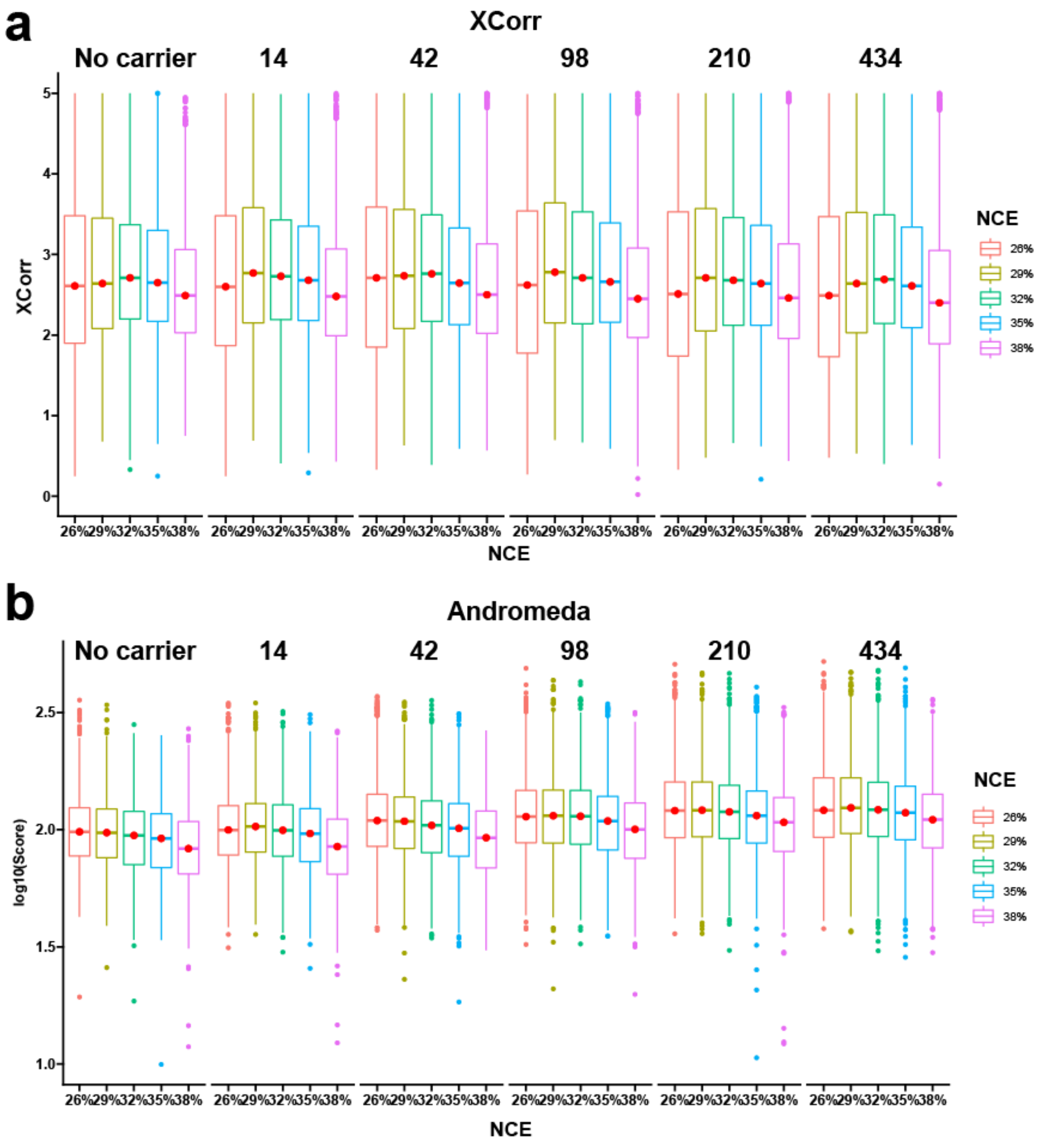


**Supplementary Figure 5 | Distribution of XCorr (a) and Andromeda (b) scores in the PSMs using different NCEs.** Samples with the following settings were selected: no carrier and carrier proteome as HEY; no carrier and carrier levels at 14 and 434; NCE at 26%, 29%, 32%, 35% and 38%. Resolution was set to 60K in all samples.

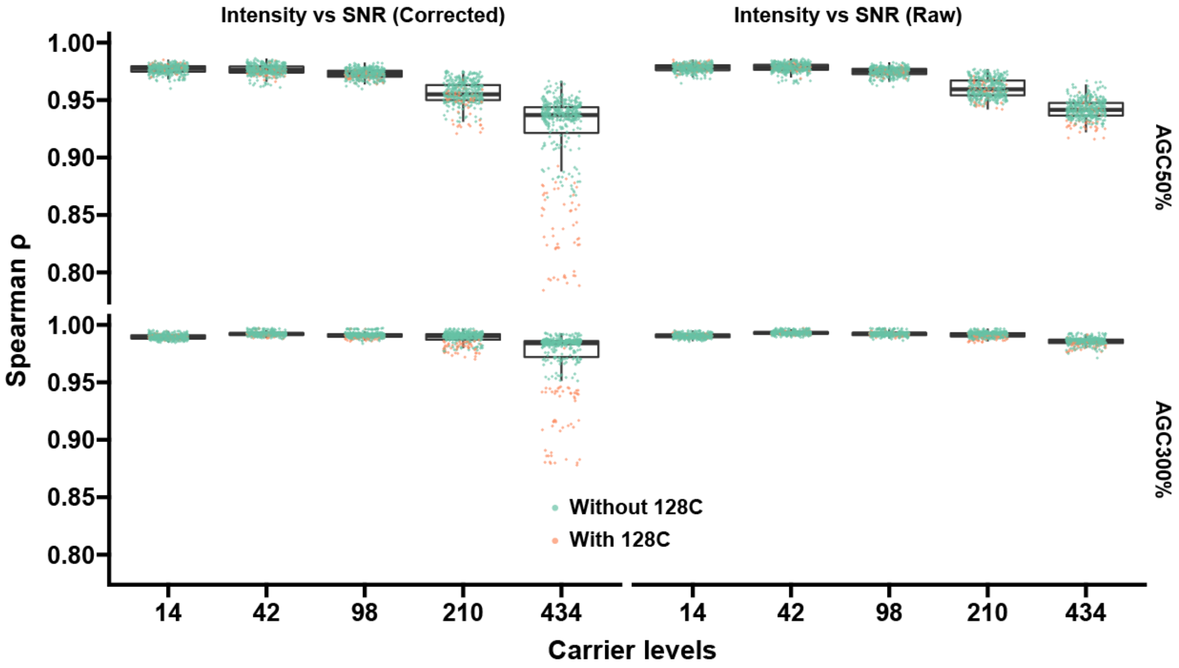


**Supplementary Figure 6 | Intensity and SNR led to different protein amount estimates.** Each point represents the Spearman ρ calculated between each two SCCs using all protein amount estimates. Within each two SCCs, SNR values were used in one channel and intensity values were used in the other channel.

## Supplementary Notes

### Supplementary Note 1

We tested 5 different carrier levels including 14x, 42x, 98x, 210x and 434x as they can increase total MS1 signal levels at 2x, 4x, 8x, 16x, 32x. For loading amounts, we tested 50pg, 100pg and 200pg per single-cell channel using human peptides as reference. Each condition was sampled in MS as duplicated. For AGC targets, we tested AGC50% and AGC300%. The normalized base (AGC100%) is 1e5 in an Orbitrap detector in MS2 scans. In addition, we also tested different NCEs (26%, 29%, 32%, 35% and 38%) at resolution 60K and different resolutions (30K, 45K, 60K and 120K) at NCE 32%. Fill time was set as auto in all runs and consequently the maximal fill time of each scan was either 119ms or 249ms.

### Supplementary Note 2

In TMTpro reagents, channel 126 and C-type mass tags have +15N impurities while N-type mass tags have -15N impurities (**Supplementary Table.SN2.1**). Despite the low proportions of these impurities, they are unneglectable when significant differences exist between related channels. For instance, the 434x CPC is expected to have 134.54% of impurities on channel 127N in our case.

| *Mass Tag* | *-2x13C* | *-13C-15N* | *-13C* | *-15N* | *Main* | *+15N* | *+13C* | *+15N+13C* | *+2x13C* |
|---|---|---|---|---|---|---|---|---|---|
| *126* | 0 | 0 | 0 | 0 | 100 | 0.31 | 8.9 | 0.03 | 0.31 |
| *127N* | 0 | 0 | 0 | 0.17 | 100 | 0 | 9.98 | 0 | 0.32 |
| *127C* | 0 | 0 | 0.93 | 0 | 100 | 0.35 | 8.63 | 0.01 | 0.27 |
| *128N* | 0 | 0 | 0.95 | 0.79 | 100 | 0 | 8.38 | 0 | 0.26 |
| *128C* | 0.04 | 0 | 1.31 | 0 | 100 | 0.35 | 6.42 | 0.01 | 0.16 |
| *129N* | 0 | 0 | 1.46 | 1.28 | 100 | 0 | 6.86 | 0 | 0.15 |
| *129C* | 0.51 | 0 | 2.74 | 0 | 100 | 0.36 | 6.15 | 0 | 0.11 |
| *130N* | 0.49 | 0.02 | 2.76 | 0.62 | 100 | 0 | 5.98 | 0 | 0.11 |
| *130C* | 0.04 | 0 | 3.1 | 0 | 100 | 0.42 | 4.82 | 0.02 | 0.06 |
| *131N* | 0.04 | 0.04 | 3.09 | 1.36 | 100 | 0 | 4.75 | 0 | 0.06 |
| *131C* | 0.08 | 0 | 3.81 | 0 | 100 | 0.4 | 3.29 | 0.03 | 0.03 |
| *132N* | 0.04 | 0 | 2.84 | 0.79 | 100 | 0 | 3.51 | 0 | 0.02 |
| *132C* | 0.1 | 0 | 4.14 | 0 | 100 | 0.4 | 1.8 | 0.02 | 0 |
| *133N* | 0.36 | 0.01 | 3.64 | 0.82 | 100 | 0 | 1.94 | 0 | 0 |
| *133C* | 0.88 | 0 | 4.7 | 0 | 100 | 0.4 | 1.01 | 0 | 0 |
| *134N* | 0.4 | 0.04 | 4.92 | 0.1 | 100 | 0 | 1.05 | 0 | 0 |

**Supplementary Table.SN2.1 | Reporter ion isotopic distributions in the batch used for the whole analysis.**

To explore the actual impurities on channel 127N, we run a sample with HeLa carrier proteome labeled with channel 126. We found that channel 126 had impurities on channel 127N, 127C, 128N, 128C and 129C (**Supplementary Fig.SN2.1**). Meanwhile, impurity correction could not completely eliminate all the impurities. In fact, the +15N impurity is not only existing in TMTpro reagents, channel 126 in TMT10plex / TMT11plex reagents also has it on a smaller scale (**Supplementary Fig.SN2.2**).

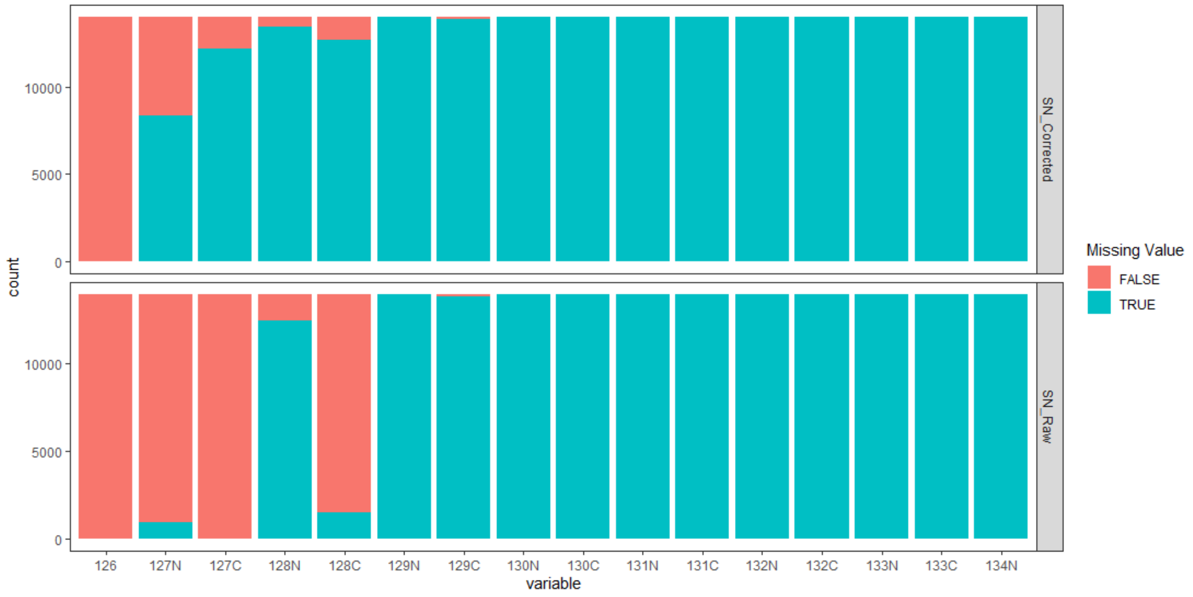


**Supplementary Fig.SN2.1 | Number of MS/MS scans with and without missing values on all TMTpro channels.** The sample was labeled with TMTpro-126 only.

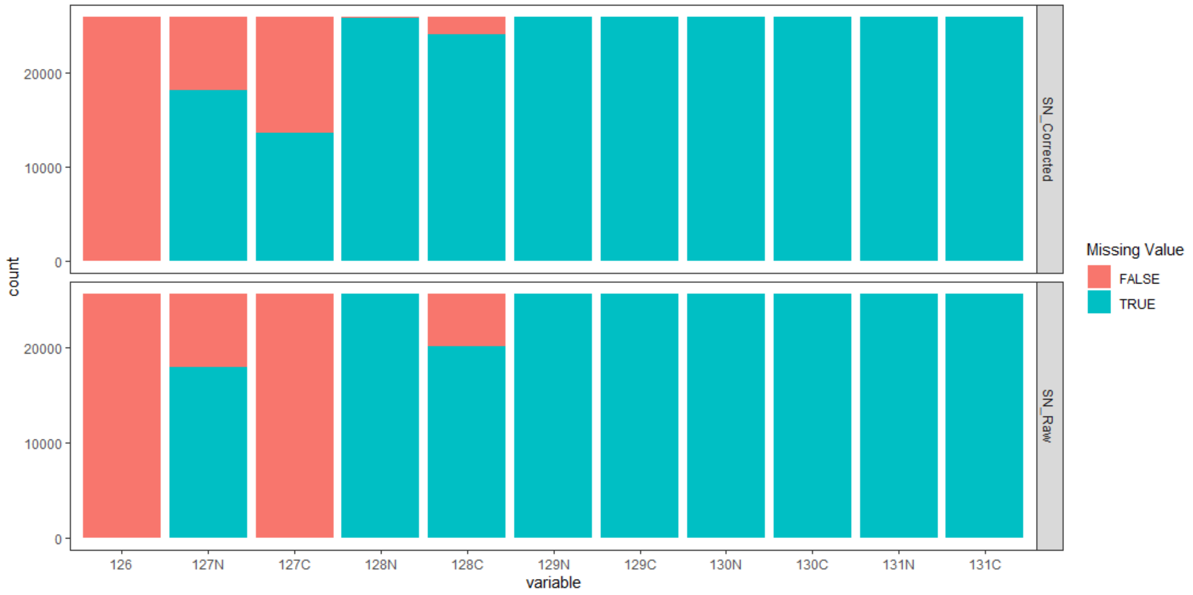


**Supplementary Fig.SN2.2 | Number of MS/MS scans with and without missing values on all TMT11plex channels.** The sample was labeled with TMT11plex-126 only.

**Supplementary Note 3**

We tested 3 different data processed software tools including Proteome Discoverer (v2.4), MaxQuant (v1.6.14.0 with adjusted TMTpro method) and SpectroMine (v2.2). We found that only Proteome Discoverer provided the option for impurity correction on +15N channels.

**Supplementary Note 4**

From the 14 SCCs, we calculated binary logarithm ($\log_2 n$) ratios including, 131C/132N, 131C/132N, 131C/132N and 131C/132N for yeast and human PSMs, 128N/128C, 129N/130C, 129C/131C and 130N/132C for E.coli PSMs. Only unique peptides to the individual species were included in the calculation.

**Supplementary Note 5**

In an Orbitrap analyzer, the number of ions is proportional to the SNR value of a peak and the intensity value is the SNR value normalized based on injection time (Proteome Discoverer 2.2 User Guide, Thermo Fisher Scientific). While actual numbers differ significantly between the SNR value and the intensity value, relative abundances on each peak are the same within each scan. Thus, relative abundances among all reporter ion peaks

are the same at PSM level regardless of whether the SNR or intensity values are used for reporter ion peaks (**Supplementary Fig.SN5.1**). Nevertheless, at protein level, protein abundances on each channel are normally based on multiple PSMs. Consequently, whether SNR or intensity values are used for quantification will generate different quantification results at protein level (**Supplementary Fig.SN5.2**).

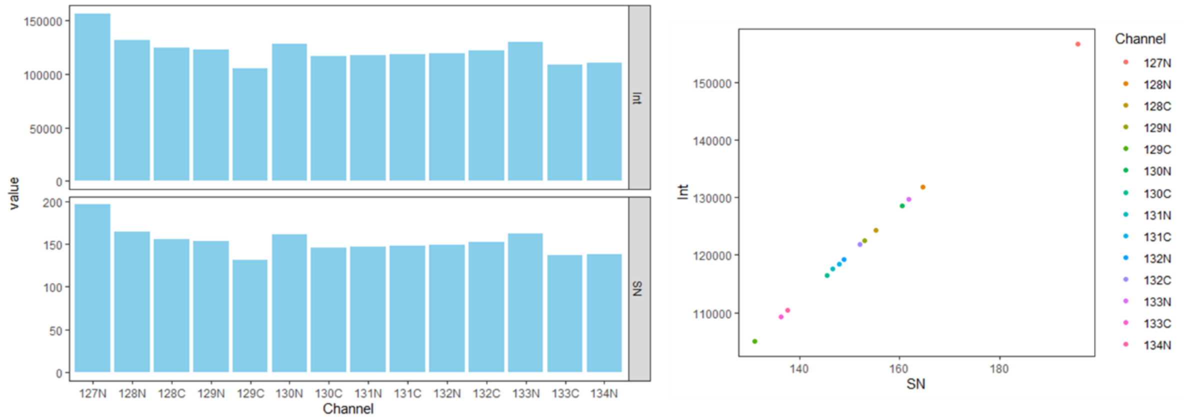


**Supplementary Fig.SN5.1 | A peptide example showing that SNR and intensity values generate identical relative abundance at PSM level.** This PSM was from peptide “VLAAVYK” in protein P04075.

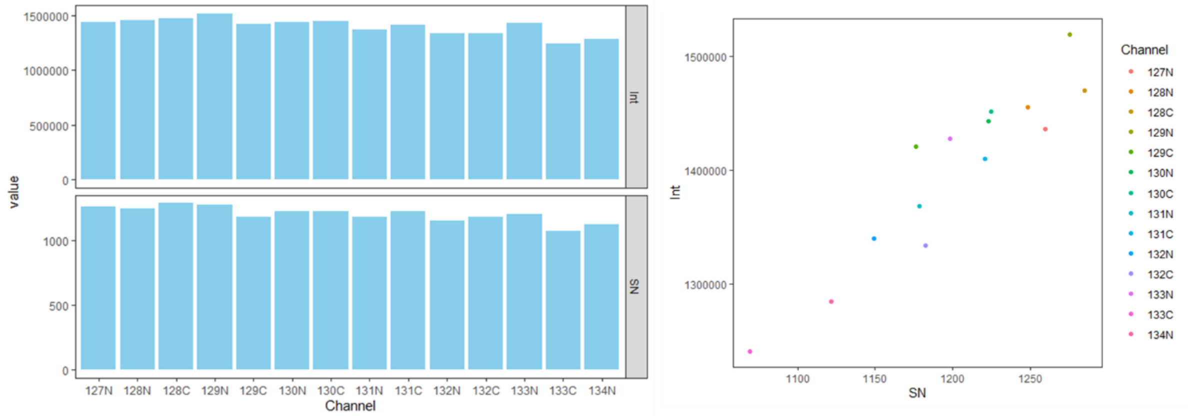


**Supplementary Fig.SN5.2 | A protein example showing that SNR and intensity values generate different relative abundance at protein level.** The protein was P04075 and it contained the PSM showed in **Supplementary Fig.SN5.1.**

### Supplementary Note 6

Since the intensity value is normalized based on ion injection time, the difference between SNR and intensity values is heavily dependent on the ion injection time. In samples with

lower AGC target settings, ion injection times in each scan are more diverse than samples with higher AGC target settings (**Supplementary Fig.SN6.1**). Thus, the correlation between SNR and intensity values is worse in samples with lower AGC target settings.

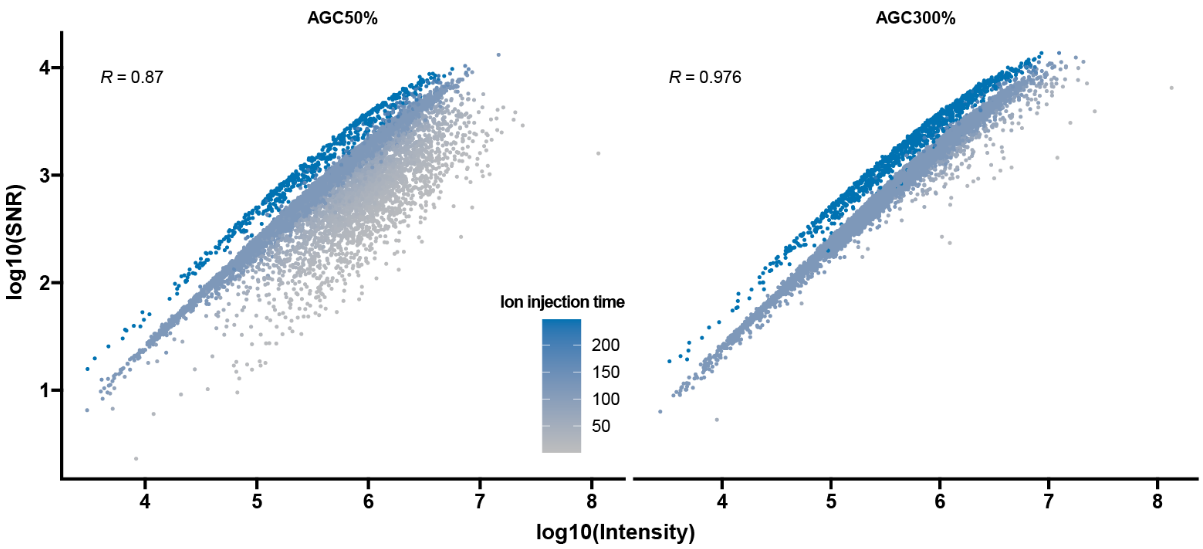


**Supplementary Fig.SN6.1 | Correlation between intensity and SNR values as reporter ion values.** Samples with the following settings were selected: carrier proteome as HEY; AGC50% and AGC300%; carrier levels at 98x. Abundance values were based on the CPC 126. The Pearson correlation coefficient was used as a measure of linear correlation.

**Supplementary Table 1 | Raw files, conditions and identifications in the study.**
