# Supplementary material for: A deeper look at carrier proteome effects for single-cell proteomics
Source: Commun Biol. 2022 Feb 22;5:150. doi: 10.1038/s42003-022-03095-4 (PMC8863851; doi:10.1038/s42003-022-03095-4)
Supplement: Supplementary file 3 — Description of Additional Supplementary Files [file 42003_2022_3095_MOESM3_ESM.pdf]

Description of Additional Supplementary Files

File name: Supplementary Data 1

Description: Source data.

File name: Supplementary Data 2

Description: Detailed description of samples.
